# Intraocular Lens Power Calculation after Corneal Refractive Surgery

**Published:** 2012-01

**Authors:** Mohammad-Ali Javadi, Sepehr Feizi, Parviz Malekifar

**Affiliations:** Ophthalmic Research Center, Shahid Beheshti University of Medical Sciences, Tehran, Iran

**Keywords:** Refractive Surgery, Intraocular Lens Power Calculation, Phacoemulsification

## Abstract

**Purpose:**

To report refractive outcomes following phacoemulsification (PE) and posterior chamber intraocular lens (PCIOL) implantation in eyes with previous corneal refractive surgery.

**Methods:**

In this retrospective comparative study, 18 consecutive eyes of 14 patients with previous keratorefractive surgery for myopia including photorefractive keratectomy (PRK, 6 eyes; 33.3%) and laser in situ keratomileusis (LASIK, 12 eyes; 66.7%) underwent PE+PCIOL. Computerized corneal topography was employed to determine the flattest keratometric reading within the 3-mm central zone. This value was inserted into the Sanders-Retzlaff-Kraff/T (SRK/T) formula to calculate IOL power. IOL power selected for implantation was 1 D greater than the calculated value described above.

**Results:**

Mean age and follow-up period were 54.1±11.5 years and 29.9±26.3 months, respectively. Mean implanted lens power was 18.56±3.86 D which was not significantly different from mean back-calculated IOL power for target refraction (19.04±4.16 D) (P=0.28). There was no significant difference between mean target refraction (−0.94±0.52 D) and achieved postoperative spherical equivalent refractive error (−0.62±1.06) at final follow-up (P=0.28). The achieved spherical equivalent refractive error was within ±0.50 D of intended refraction in 8 (44.4%) eyes, within ±1.0 D in 11 (61.1%) eyes, and within ±2.0 D in 16 (88.9%) eyes. In a subgroup of patients (5 eyes) with complete pre-refractive surgery data, the difference between post-refractive surgery keratometry method and all other methods (P=0.02) and between the current method and the Feiz-Mannis method (P=0.01) was statistically significant.

**Conclusion:**

The method suggested herein is simple and independent of pre-refractive surgery data with results comparable to other commonly used methods.

## INTRODUCTION

Patients with previous corneal refractive procedures such as radial keratotomy (RK), photorefractive keratectomy (PRK), and laser in situ keratomileusis (LASIK) encounter certain problems when they require cataract surgery and intraocular lens (IOL) implantation. They understandably have high expectations for uncorrected visual acuity, similar to what they experienced after refractive surgery. This causes a significant challenge because IOL power calculations after refractive surgery are known to be less predictable in these eyes as compared to eyes with virgin corneas.^1^–^5^

Sources of unsatisfactory IOL calculations after laser refractive surgery include instrument errors, index of refraction errors, and formula errors.^6^,^7^ A significant source of instrument error is the fact that most keratometers measure central corneal radius of curvature in the 2.5 to 3.2 mm zone and assume a sphero-cylindrical cornea, an assumption that is incorrect after myopic refractive surgery,^5^,^8^ leading to overestimation of corneal refractive power by 15% to 25% and consecutively, hyperopic outcomes.^1^,^9^ Furthermore, when the anterior but not the posterior corneal surface has been modified as following myopic laser refractive surgery (but not after RK), errors due to an altered index of refraction occur because the relationship assumed in keratometers (1.3375) between the two surfaces is no longer applicable.^2^,^5^ Finally formula errors occur because the widely used third generation IOL formulas, such as the Holladay, Hoffer Q and SRK/T, use corneal power to predict the pseudophakic anterior chamber depth. Since the cornea becomes flattened after myopic laser surgery, the anterior chamber is incorrectly assumed to be shallow, while it actually has remained negligibly altered.^7^ Therefore, these third generation formulas calculate a falsely shallow pseudophakic anterior chamber depth, leading to underestimation of IOL power. This IOL power underestimation stems from the overestimated corneal refractive power.^10^ These sources of error culminate in what has been termed as “hyperopic surprise” commonly observed after cataract surgery in myopic excimer laser-treated eyes.

Several methods have been suggested to improve the accuracy of IOL power calculations^2^,^11^–^14^ however, none of them appear to be adequately precise. Additionally, such diversity can easily generate confusion rather than accuracy. In the present study, we present the outcomes of a simple method of IOL power calculation after refractive surgery and evaluate its accuracy in comparison with other commonly used methods, namely the clinical history,^11^ Feiz-Mannis,^12^ and corneal power bypassing^13^ methods.

## METHODS

This retrospective comparative study was performed on 18 eyes of 14 subjects including 11 male patients who had undergone keratorefractive surgery for myopia including PRK and LASIK. All subjects had developed visually significant cataracts and underwent phacoemulsification (PE) and posterior chamber IOL (PCIOL) implantation from March 2001 to February 2010. Pre-refractive surgery data were available in only 5 eyes. All patients had undergone a comprehensive ophthalmologic examination before cataract surgery including determination of uncorrected and best-spectacle corrected visual acuity (UCVA and BSCVA), manifest refraction, keratometry using a manual Javal-Schiötz keratometer (Topcon, Capelle a/d IJssel, Netherlands), slit lamp and dilated fundus examination, and intraocular pressure measurement.

A computerized corneal topography analysis (TMS-1 Topographic Modeling System, version 1.61; Computed Anatomy Inc., NY, USA) was used to determine the flattest keratometric reading within the 3-mm central zone. This value was employed for IOL power calculation using the SRK-T formula in all cases. Axial length was determined by contact A-scan ultrasound biometry (Storz Omega Compu-Scan Biometric Ruler, Storz International, St Louis, MO, USA). The power of the implanted IOL was 1 D greater than that calculated.

### Surgical Technique

PE+PCIOL was performed under retrobulbar anesthesia in all patients. A 2.8 mm clear corneal tunnel, 1.5 to 2.0 mm in length, was made. After injection of a dispersive ophthalmic viscosurgical device (Coatel, Bausch & Lomb, Waterford, Ireland), a 5.0 to 5.5 mm central continuous capsulorrhexis was created and PE was performed using the divide and conquer technique. This step was followed by cortical cleanup and implantation of a foldable one-piece monofocal IOL (AcrySof SA60AT, A constant 118.4, Alcon Laboratories Inc., Fort Worth, TX, USA) within the capsular bag using the C-type cartridge (Alcon Laboratories Inc.). At the conclusion of surgery, the anterior chamber was formed and the clear corneal incision was hydrated to become self-sealed.

Postoperatively, sulfacetamide 10% eye drops every 6 hours and betamethasone 0.1% eye drops every 3 hours were prescribed. The antibiotic drops were discontinued after 10 days, while the corticosteroid drops were tapered over 4 to 6 weeks. Follow-up examinations were scheduled 1, 3, and 7 days, 1, 3, and 6 months, and every 6 months thereafter. During follow-up examinations, UCVA, BSCVA, keratometry, manifest refraction, and intraocular pressure were evaluated.

### Statistical Analysis

Data was analyzed using the SPSS (version 15) statistical software (SPSS Inc., Chicago, IL, USA). Normality was checked by the Kolmogorov-Smirnov test and normally distributed data were expressed in mean ± standard deviation. The Student t-test was used to compare target and achieved postoperative refraction (the latter was expressed in spherical equivalent). One-way ANOVA was applied to compare IOL powers calculated by different methods. P-values less than 5% were considered as statistically significant.

Intraocular lens power required for achieving the target refraction was back-calculated using stable post-cataract surgery manifest refraction and implanted IOL power as discussed by Olsen^15^ and Aramberri^10^ (Pt = Pi +1.5×R, where Pi = power of implanted IOL, R = difference between target and postoperative manifest refraction, and Pt = power required for achieving target refraction). This value was compared with the implanted IOL power.

In a subgroup of patients with available pre-refractive surgery data, the historical,^11^ Feiz-Mannis,^12^ and corneal power bypassing^13^ methods were used to determine refractive corneal power. These derived corneal powers were then used in the SRK-T formula to determine IOL power. The results were compared with the implanted and back-calculated IOL powers.

## RESULTS

A total of 18 eyes of 14 patients with mean age of 54.1±11.5 (range, 33–73) years were operated. Mean follow-up duration was 29.9±26.3 (range, 3–92) months. Mean interval between refractive and cataract surgery was 70.7±35.0 (range, 18–153) months. The type of refractive surgery was LASIK in 12 eyes and PRK in 6 other eyes.

Before cataract surgery, mean keratometric readings by manual keratometry was 40.72±2.36 D (range, 35.75 to 43.65 D) which was significantly steeper than the applied values as determined by topography (39.79±3.0 D, range, 33.50 to 43.50 D, P=0.01).

Mean UCVA and BSCVA values after cataract surgery were 0.33±0.15 (range, 0.10 to 0.60) logMAR and 0.19±0.13 (range, 0 to 0.4) logMAR, respectively.

Mean target refraction and achieved postoperative spherical equivalent at final follow-up were −0.94±0.52 (range, −1.75 to +0.26) D and −0.62±1.06 (range, −3.0 to +1.38) D, respectively. Mean difference between these values was −0.36±1.23 (range, −2.36 to +2.06 D, P=0.28) and mean absolute difference was 1.02±0.74 (range, 0.15 to 2.36) D. The achieved spherical equivalent refraction was within ±0.50 D of intended refraction in 8 (44.4%) eyes, within ±1.0 D in 11 (61.1%) eyes, and within ±2.0 D in 16 (88.9%) eyes.

Mean power of the implanted IOL was 18.56±3.86 (range, 9.0 to 24.0) D and mean back-calculated IOL power for target refraction was 19.04±4.16 (range, 5.97 to 24.70) D (P=0.28). Mean calculated IOL power based on post-refractive surgery simulated keratometry (17.56±4.87 D; range, 4.0 to 23.0 D) was significantly lower than mean power of the implanted IOLs (P=0.01) and back-calculated IOL power (P=0.02).

**Figure 1.**
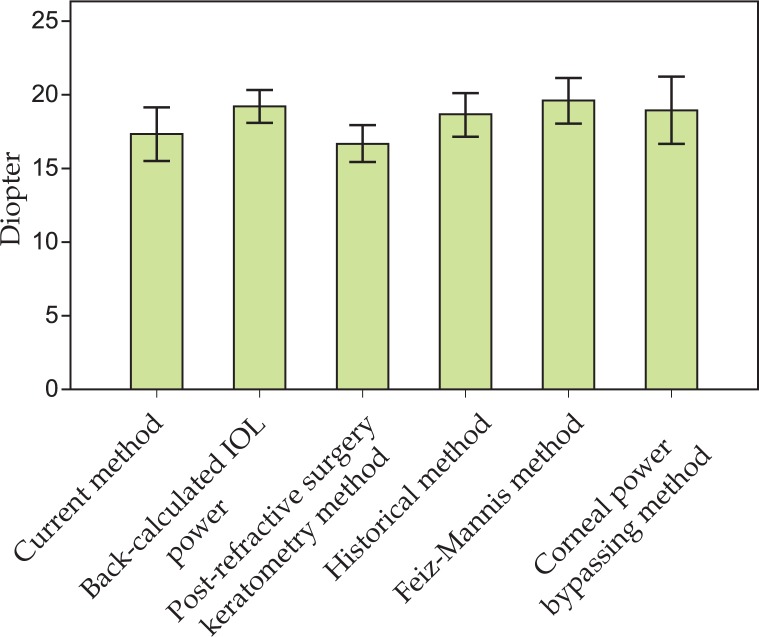
Results of different methods of intraocular lens power calculation after corneal refractive surgery in 5 eyes.

Data before, immediately after refractive surgery, after stabilization of refraction and before development of cataracts was available in only 5 eyes (Table 1), of which 4 had undergone LASIK. Spherical equivalent refraction was reduced by 4.91±1.65 D after refractive surgery in this subgroup of eyes (P=0.01). In these eyes, pre-refractive surgery data was available and it was possible to compare the accuracy of our method with other ones including the clinical history, Feiz-Mannis, and corneal power bypassing methods (Fig. 1). IOL power calculation was lowest with the post-refractive surgery keratometry method and highest with the Feiz-Mannis method. The post-refractive surgery keratometry method was significantly different from all other methods (P=0.02). The difference between the current method and the Feiz-Mannis method was also statistically significant (P=0.01). Differences between the current method and other methods were not statistically significant.

### Representative Case

A 46-year-old man underwent PE+PCIOL in his left eye because of a visually significant posterior subcapsular cataract 5 years after bilateral myopic PRK. Pre-PRK refraction and simulated keratometry reading had been −3.5–1.25×170 and 44.25×170°/46.25×80°. After PRK, refraction was −0.25–0.75×170°, simulated keratometric readings were 42.0×150°/43.5×60° and antero-posterior axial length was 25.20 mm. Target refraction after cataract surgery was set for emmetropia. IOL powers were calculated by different methods as follows:

#### Post-Refractive Surgery Keratometry Method

Using the mean post-PRK keratometry value (42.75 D), IOL power was calculated to be 16.50 D for target refraction.

#### Clinical History Method^11^

A reduction of 3.5 D in the pre-PRK spherical equivalent refractive error was subtracted from the pre-PRK mean keratometry reading (45.25 D) to achieve a postoperative keratometry value of 41.75 D; calculated IOL power for the target refraction was 17.5 D.

#### Feiz-Mannis Method^12^

According to the nomogram introduced by Feiz et al,^12^ 1.85 D was added to the IOL power calculated by the post-PRK keratometry method (16.5 D) for a 3.5-D reduction in spherical equivalent refractive error induced by PRK. This method yielded an IOL power of 18.5 D.

#### Corneal Power Bypassing Method^13^

This method assumes that the patient never had PRK and uses pre-PRK keratometric values (44.25 and 46.25 D) and postoperative axial length (25.20 mm). However, the amount of reduction in pre-PRK spherical equivalent refraction (3.5 D) is considered as the target refraction. Using this method which bypasses the post-PRK corneal power, IOL power was calculated 18.5 D.

#### Current Method

The flattest meridian within 3-mm central zone (42.92 D) was entered in the SRK-T formula which resulted in a power of 16.5 D for the target refraction. A 17.5 D PCIOL was implanted. At final follow-up, 4 years after cataract surgery, manifest refraction was +0.25–0.50×155, UCVA was 20/25 and BSCVA was 20/20. The postoperative spherical equivalent was zero, indicating that a suitable power had been chosen.

## DISCUSSION

Several methods have been developed to compensate for errors caused by inaccuracy in corneal power measurement after keratorefractive surgery.^2^,^11^–^14^ These methods include the clinical history method,^11^ the Shammas refraction-derived corrected K value,^14^ IOL power adjustment according to the amount of laser treatment,^2^ the Feiz-Mannis method,^12^ and a method that bypasses corneal power.^13^ This variety however, is confusing. Additionally, there are drawbacks to each technique. These methods require knowledge of preoperative corneal power and the amount of myopic correction obtained by refractive surgery which may not be readily available. The contact lens method depends on obtaining an accurate refraction, which may not be possible in a patient with a visually significant cataract.

In this study, we evaluated the results of IOL power calculation using a simple method in comparison with other commonly used methods. In the current scheme, the flattest keratometric reading within the 3-mm central zone was determined using topography and the value was used in the SRK/T formula. As indicated in the current study, the flattest keratometry readings by topography are more accurate for calculating IOL power than values obtained by manual keratometry which tend to overestimate curvature.^18^

The choice of the SRK-T formula was based on its accuracy in long eyes.^19^ Therefore, patients would have become hyperopic if the results by the SRK-T formula had been used without adding 1 D. This undercorrection may stem from inaccurate keratometry index used by the topographer and/or shallow anterior chamber depth wrongly considered by the SRK-T formula due to flattened corneal curvature.

Results of the present study indicate that postoperative refractive error and implanted IOL power did not significantly differ from target refraction and back-calculated IOL power, respectively. Additionally, postoperative spherical equivalent refraction was within ±1.00 D and ±2.0 D of intended refraction in 61.1% and 88.9% of eyes, respectively. These results are in good agreement with refractive outcomes reported after cataract surgery in patients with previous refractive surgery. Furthermore, our results are very close to those reported in studies on patients who have not undergone excimer laser surgery.^20^–^22^

Walter et al^13^ described a method for calculating IOL power after LASIK in which pre-LASIK keratometric values and post-LASIK axial length were placed in the Holladay formula and the target refraction was set at pre-LASIK manifest refraction. Using this method which is independent of the inaccurate post-LASIK corneal power, they reported a mean postoperative spherical equivalent refractive error of +0.03±0.42 D, ranging from −0.625 to +0.75 D. Masket and Masket^2^ found that the amount of treated myopia is the chief corrective factor in determining the most accurate IOL power calculation and developed a simple regression formula for IOL power adjustment. They noted that 3 D of laser correction will alter IOL power by approximately 1 D. Using this correction factor for 30 eyes, they reported that more than 90% of operated eyes had postoperative refraction within ±0.5 D of target refraction with mean error of −0.15±0.29 (range, −0.75 to +0.5) D. Feiz et al^12^ determined the IOL power for emmetropia before LASIK supposing that the patient had had no corneal refractive procedure. Then, they predicted the power by subtracting the change in preoperative myopia from this value and developed a nomogram to adjust IOL power based on the amount of change in refractive error.

Using Orbscan II to measure the central 2mm total-mean corneal power, Arce et al^23^ reported postoperative spherical equivalent refraction of −0.52±0.79 (range, −3.12 to +1.25) D. They reached better outcomes with the SRK-T formula than with the Holladay, Hoffer-Q, and Haigis formulas in eyes with previous myopic PRK or myopic LASIK than in eyes with hyperopic LASIK. Using corneal topography or IOLMaster to measure corneal power and different methods to calculate IOL power, Savini et al^24^ reported mean difference between target refraction and spherical equivalent one month after cataract surgery to be −0.43±0.59 (range, −1.50 to +0.25) D. A similar study by McCarthy et al reported mean postoperative spherical equivalent of −0.43±0.90 (range, −2.75 to +2.75) D.^18^

In 5 eyes of our series, it was possible to calculate the IOL power backward using the clinical history, Feiz-Mannis, and corneal power bypassing method. Compared to our method, the Feiz-Mannis method yielded significantly higher IOL powers. However, there was no significant difference between IOL power calculated by the Feiz-Mannis method and back-calculated IOL power, or between the present, clinical history, and corneal power bypassing methods. This finding means that our method is at least comparable to the abovementioned methods in term of accuracy and can be used interchangeably. However, considering the small number of eyes in this subgroup, it is difficult to reach a definite conclusion. Further studies with larger sample size are required to reach a reliable conclusion.

In summary, the majority of patients obtained satisfactory visual outcomes using the present method which does not depend on pre-refractive surgery data. However, a significant percentage had unacceptable postoperative refractive errors. Patients should have realistic expectations and they should be informed about the possibility of needing, glasses, IOL exchange or further laser refractive surgery.

## Figures and Tables

**Table 1.** Refraction and mean keratometry readings before and after refractive surgery in 5 eyes

**Parameter**	**Before refractive surgery Mean ± SD (range)**	**After refractive surgery Mean ± SD (range)**	**P-value**
Spherical equivalent (D)	−4.94 ± 1.71 (−7.50 to −2.5)	−0.03 ± 0.50 (−0.63 to +0.38)	0.01
Mean Keratometry (D)	44.50 ± 0.55 (44.0 to 45.25)	39.59 ± 1.89 (37.25 to 41.90)*	0.002

Mean keratometry after refractive surgery was calculated using the historical method

## References

[1] Seitz B, Langenbucher A, Nguyen NX, Kus MM, Küchle M (1999). Underestimation of intraocular lens power for cataract surgery after myopic photorefractive keratectomy. Ophthalmology.

[2] Masket S, Masket SE (2006). Simple regression formula for intraocular lens power adjustment in eyes requiring cataract surgery after excimer laser photoablation. J Cataract Refract Surg.

[3] Koch DD (2006). New options for IOL calculations after refractive surgery. J Cataract Refract Surg.

[4] Langenbucher A, Haigis W, Seitz B (2004). Difficult lens power calculations. Curr Opin Ophthalmol.

[5] Hamilton DR, Hardten DR (2003). Cataract surgery in patients with prior refractive surgery. Curr Opin Ophthalmol.

[6] Hoffer KJ (2009). Intraocular lens power calculations after previous laser refractive surgery. J Cataract Refract Surg.

[7] Haigis W (2008). Intraocular lens calculation after refractive surgery for myopia: Haigis-L formula. J Cataract Refract Surg.

[8] Rosa N, Capasso L, Lanza M, Furgiuele D, Romano A (2004). Reliability of the IOLMaster in measuring corneal power changes after photorefractive keratectomy. J Cataract Refract Surg.

[9] Chan CC, Hodge C, Lawless M (2006). Calculation of intraocular lens power after corneal refractive surgery. Clin Experiment Ophthalmol.

[10] Aramberri J (2003). Intraocular lens power calculation after corneal refractive surgery: double-K method. J Cataract Refract Surg.

[11] Holladay JT (1997). Cataract surgery in patients with previous keratorefractive surgery (RK, PRK, and LASIK). Ophthalmic Practice.

[12] Feiz V, Mannis MJ, Garcia-Ferrer F, Kandavel G, Darlington JK, Kim E (2001). Intraocular lens power calculation after laser in situ keratomileusis for myopia and hyperopia: a standardized approach. Cornea.

[13] Walter KA, Gagnon MR, Hoopes PC, Dickinson PJ (2006). Accurate intraocular lens power calculation after myopic laser in situ keratomileusis, bypassing corneal power. J Cataract Refract Surg.

[14] Shammas HJ, Shammas MC, Garabet A, Kim JH, Shammas A, LaBree L (2003). Correcting the corneal power measurements for intraocular lens power calculations after myopic laser in situ keratomileusis. Am J Ophthalmol.

[15] Olsen T (2007). Calculation of intraocular lens power: a review. Acta Ophthalmol Scand.

[16] Wang L, Booth MA, Koch DD (2004). Comparison of intraocular lens power calculation methods in eyes that have undergone LASIK. Ophthalmology.

[17] Sónego-Krone S, López-Moreno G, Beaujon-Balbi O, Arce CG, Schor P, Campos M (2004). A direct method to measure the power of the central cornea after myopic laser in situ keratomileusis. Arch Ophthalmol.

[18] McCarthy M, Gavanski GM, Paton KE, Holland SP (2011). Intraocular lens power calculations after myopic laser refractive surgery: a comparison of methods in 173 eyes. Ophthalmology.

[19] Hoffer KJ (2000). Clinical results using the Holladay 2 intraocular lens power formula. J Cataract Refract Surg.

[20] Savini G, Barboni P, Carbonelli M, Hoffer KJ (2009). Accuracy of Scheimpflug corneal power measurements for intraocular lens power calculation. J Cataract Refract Surg.

[21] Olsen T (2007). Improved accuracy of intraocular lens power calculation with the Zeiss IOLMaster. Acta Ophthalmol Scand.

[22] Narváez J, Zimmerman G, Stulting RD, Chang DH (2006). Accuracy of intraocular lens power prediction using the Hoffer Q, Holladay 1, Holladay 2, and SRK/T formulas. J Cataract Refract Surg.

[23] Arce CG, Soriano ES, Weisenthal RW, Hamilton SM, Rocha KM, Alzamora JB (2009). Calculation of intraocular lens power using Orbscan II quantitative area topography after corneal refractive surgery. J Refract Surg.

[24] Savini G, Hoffer KJ, Carbonelli M, Barboni P (2010). Intraocular lens power calculation after myopic excimer laser surgery: clinical comparison of published methods. J Cataract Refract Surg.

